# Objective Assessment of Endovascular Navigation Skills with Force Sensing

**DOI:** 10.1007/s10439-017-1791-y

**Published:** 2017-02-08

**Authors:** Hedyeh Rafii-Tari, Christopher J. Payne, Colin Bicknell, Ka-Wai Kwok, Nicholas J. W. Cheshire, Celia Riga, Guang-Zhong Yang

**Affiliations:** 1 The Hamlyn Centre for Robotic Surgery, Imperial College London, Level 4, Bessemer Building, South Kensington Campus, London, SW7 2AZ UK; 2 Academic Division of Surgery, Imperial College London, London, UK; 3 Department of Mechanical Engineering, The University of Hong Kong, Hong Kong, People’s Republic of China

**Keywords:** Endovascular intervention, Force sensing, Skill assessment, Haptic and navigation cues, Classification of skill, Catheter manipulation, Robotic catheterization

## Abstract

Despite the increasing popularity of endovascular intervention in clinical practice, there remains a lack of objective and quantitative metrics for skill evaluation of endovascular techniques. Data relating to the forces exerted during endovascular procedures and the behavioral patterns of endovascular clinicians is currently limited. This research proposes two platforms for measuring tool forces applied by operators and contact forces resulting from catheter–tissue interactions, as a means of providing accurate, objective metrics of operator skill within a realistic simulation environment. Operator manipulation patterns are compared across different experience levels performing various complex catheterization tasks, and different performance metrics relating to tool forces, catheter motion dynamics, and forces exerted on the vasculature are extracted. The results depict significant differences between the two experience groups in their force and motion patterns across different phases of the procedures, with support vector machine (SVM) classification showing cross-validation accuracies as high as 90% between the two skill levels. This is the first robust study, validated across a large pool of endovascular specialists, to present objective measures of endovascular skill based on exerted forces. The study also provides significant insights into the design of optimized metrics for improved training and performance assessment of catheterization tasks.

## Introduction

Numerous studies have shown the steep learning curves associated with endovascular catheterization, and that clinical outcomes are highly dependent on operator experience.^5^,^9^ However, studies on operator force and motion patterns, tool–tissue interactions, and behavioural data are very limited. The field of endovascular intervention suffers from a lack of objective and quantitative skill assessment measures.^18^ As a result, designing metrics that enable accurate and objective analysis of operator manipulation patterns and forces exerted on the vasculature within a realistic simulation environment has the potential to improve assessment and training of catheterization skills.

In order to navigate the catheters and guidewires through different arteries in the body, in practice the operator relies on a combination of haptic and visual cues, achieved by sensing the small axial forces/torques at the fingertips combined with 2D fluoroscopy imaging. Catheter navigation is achieved through a combination of insertion, retraction, and twist at the proximal end. Factors that can contribute to difficulties and increase the risk of procedural complications include catheter instability, operator experience, as well as vessel tortuosity and angulation which cause difficulties in steering devices and reaching the target site.^5^,^10^,^25^ Understanding the skill-related behaviour patterns of operators, as well as quantification of contact forces resulting from tool–tissue interactions, can provide important insights into potential intra-procedural risks including thrombosis, dissection and perforation, especially for weakened and diseased vessel walls.^6^

The training of endovascular skills has thus far relied on different tools including synthetic models, animals, cadavers, and virtual reality (VR) simulators.^11^ However, endovascular skill assessment suffers from a lack of uniformly accepted and objective measures and credentialing guidelines that take into account force and motion-related measures of manipulation, the devices and the vasculature. Due to the potential of VR simulators as endovascular assessment and training tools that can combine both quantitative and qualitative performance metrics, they have witnessed a growing interest in recent years.^2^ These include full body mannequins such as the VIST^®^-Lab (Mentice AB, Sweden) which consists of simulated instruments, and performance metrics such as contrast volume and fluoroscopy time.^3^ Other simulators provide pre-procedure rehearsal and simulated training of different procedures with added haptic feedback.^4^ Thus far quantitative information on operator–tool interactions, tool–tissue interactions, and skill-related behaviour patterns is very limited. Some studies have looked at motion profiles of interventionalists by tracking their finger motion in animal models and simulators.^22^ Other research has studied catheter dynamics by using specialized sensors to measure forces and torques required to overcome sheath or vasculature friction.^24^ For visualization of the contact between catheter/guidewires and vascular models, photoelastic stress analysis has been used, combined with tracking operator hand motions and proximal catheter motion, to provide technical metrics for measurement of skill.^8^,^23^ Other clinical research has relied on 2D catheter tip tracking (from fluoroscopy images) for skill assessment based on the catheter path-length.^21^

In recent years, the growing interest in robotic surgical systems and simulators has led to an increased demand for more objective measures of skill evaluation. Force sensing platforms have been explored as a means of measuring tool–tissue interaction forces exerted by laparoscopic tools or robotic instruments within task boards or box trainers, proposing contact force measurements as valid objective measures of assessing skill for surgical training.^7^ For endovascular procedures, most of the interest in force sensing technologies has been in the field of cardiac electrophysiology for measuring contact forces at the catheter tip. These are used to avoid excessive forces as well as maintaining contact between catheter electrodes and the myocardial wall during cardiac ablation. Different commercial solutions have been proposed, including the TactiCath^®^ catheter (Endosense SA, Geneva, Switzerland) which can measure the magnitude and angle of the force applied at the tip,^20^ as well as the IntelliSense^®^ system incorporated with the robotic catheterization platform Sensei^®^X (Hansen Medical, Mountain View, CA, USA).^12^ For peripheral vascular procedures, studies have attempted to show the significance of providing additional force feedback towards enhancing the tactile cues felt by operators whilst reducing the potential intra-procedural risks,^13^ however catheter force sensing technologies still remain in the research stage. This is due to miniaturization issues and the higher cost of integration associated with the smaller size of these catheters. Furthermore, there is a need for measuring side, and not just tip forces, due to the interactions of the entire catheter shape with the vascular anatomy. As a result no established commercial force sensing solutions exist as of yet. Information on interaction forces between catheters, guidewires, endovascular tools and the anatomy is very limited.

This paper proposes two platforms for measuring both proximal tool forces applied by operators as well contact forces resulting from catheter–tissue interactions, and using this information as a means of providing objective quantitative metrics of operator skill and surgical performance. Skill related navigation strategies are compared between different experience levels performing multiple catheterization tasks within a realistic endovascular simulation environment. Different performance metrics relating to operator force/torque patterns, quality of catheter tip motion, as well as magnitude, impact and duration of contact forces exerted on the vasculature were extracted, so as to gain an understanding of the underlying skills that contribute to overall operator performance. The original design of the force sensing platforms and preliminary results on a few subjects have been reported previously.^16^,^17^ This study continues that work with extensive validation on a larger pool of experienced endovascular surgeons and interventional radiologists. They perform various catheterization tasks within complex anatomical settings of both the abdominal and thoracic aorta, with clinical complications ranging from aneurysms to tortuous arteries. The experimental setup was improved to create a more realistic endovascular simulation environment and enable automatic synchronization between the different sensing modalities. A more thorough set of performance metrics has been extracted from the measurements to further highlight the underlying characteristics of skill, and support vector machine (SVM) classification is used on the force signals to classify skill level for the different catheterization tasks. This is the first work to propose proximal and distal force sensing as objective measures for endovascular skill assessment, whilst providing significant insights into designing improved metrics for evaluation of catheterization skills.

## Materials and Methods

### Proximal Force Sensing

This section provides details of the force-torque (F/T) sensor design attached to the proximal end of the catheter, together with a position sensor at the catheter tip, for relating tool forces applied by operators to catheter tip motion and overall operator performance.

**Figure 1:**
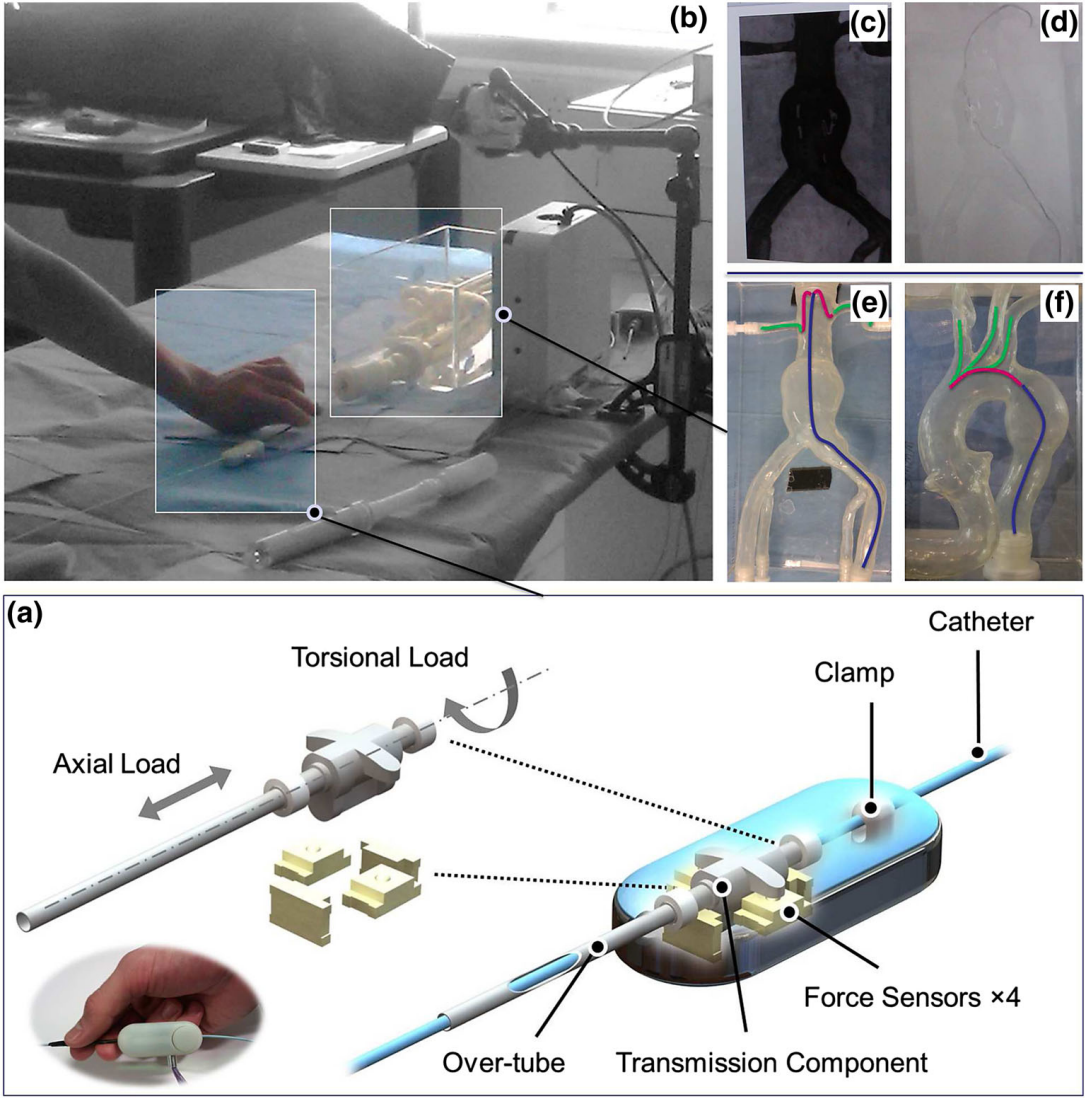
The F/T sensor mounted on the catheter with an exploded view of the 4 force sensors and the transmission component (a), the vascular model (b), simulated fluoroscopy and DSA images used for guidance (c, d), and the two phantoms with the three procedural phases (e, f).

#### Proximal Sensor Design

In order to assess the forces and torques exerted by the interventionalist on to the catheter, a proximal F/T sensing unit was devised (Fig. 1a).^17^ The sensing unit incorporates a flexible co-axial over-tube that the operator grasps instead of the catheter itself. This over-tube is coupled to a mechanical assembly incorporating four force sensors (FSS1500NS, Honeywell) that measure axial (push/pull) and torsional (clockwise/counterclockwise twist) loads. The sensing unit itself is designed to be unobtrusive to the operator; it is compact and lightweight to avoid interfering with the catheter dynamics during manipulation. The force sensors were calibrated against a Nano17 F/T sensor (ATI Industrial Automation Inc., USA). A spring-loaded clamp allows the sensing unit to clasp the catheter, thereby it can be positioned anywhere along the length of the catheter that is comfortable to the operator.

#### Experimental Setup

A phantom study was devised in order to related the forces applied at the proximal end to catheter tip motion and dynamics. Two silicone-based, transparent, anthropomorphic phantoms (Elastrat Sarl, Geneva, Switzerland), consisting of (1) an ***abdominal aneurysm model*** with a tortuous iliac artery and (2) an ***aortic arch model*** with an aneurysm in the descending aorta, were filled with water and used for this study (Fig. 1b). In order to simulate 2D fluoroscopy (the standard intra-operative guidance technique), live images obtained from a camera mounted above the phantoms were processed using contrast, brightness, and color adjustment. This enabled removal of the contours of the vessels and prevention of depth perception while still allowing visualization of the catheter and guidewires. Furthermore, pre-processed static images of each of the vascular models, obtained at different angles, were used for simulating 2D digital subtraction angiography (DSA) road-maps. Both the live simulated fluoroscopy and DSA road-maps were projected onto a monitor, to be used by the operators for navigation (Figs. 1c and 1d).

Information regarding catheter tip motion was extracted by integrating a 5-DoF electromagnetic (EM) position sensor (Aurora, Northern Digital Inc. CA) at the tip of a 5F conventional shaped catheter. The sensor consisted of a $\phi 0.5 \,{\text{mm}} \times 8\,{\text{mm}}$ sensor coil and was selected for its small size and flexibility, so as to preserve the catheter’s original size and shaped tip whilst minimizing the effects on catheter dynamics and natural motion. In order to obtain direct measurements from the catheter tip while protecting the sensor from water, the sensor was attached to the tip of the catheter using medical-grade bio-compatible thin-walled heat shrink tubing (wall thickness = 0.0127 mm, Vention Medical Inc. USA). Since the sensor origin is not located at the tip of the sensor, a pivot calibration was performed to find the offset between the sensor origin and the tip of the sensor. This was performed by fixing the tip of the sensor on a custom-designed pivot block and changing the orientation of the sensor, thereby obtaining 800 samples at different orientations. The results showed an offset of 3.60 mm with an RMS error of 0.57 mm.

Acquisition and synchronization of the force data, EM position data, and the video feed was provided by multi-threaded custom software written in C++. The software utilized UDP communication to simultaneously stream the force data through LabVIEW using an acquisition card (NI-USB6009, National Instruments Corp., USA. Frequency = 25 Hz), the EM data (Aurora NDI, CA. Frequency = 40 Hz) and the video feed, which was processed, displayed and recorded using the OpenCV library (Open Source Computer Vision. 30 fps). The software output consisted of a recorded video file and a synchronized data file containing the force data, EM recordings and video frames with corresponding time stamps.

Five endovascular tasks were defined for this study: cannulation of the left subclavian artery (LSA), the left common carotid artery (LCCA), and the right common carotid artery (RCCA) in the aortic arch model with an aneurysm, as well as cannulation of the left renal artery (LRA) and the right renal artery (RRA) in the abdominal aneurysm model. Cannulation of each of the arteries was performed multiple times (*n* = 48) across 16 operators of varying endovascular experience who were separated into two groups: 6 experienced operators (*n* = 18, experienced vascular surgeons and interventional radiologists who had performed >100 endovascular procedures) and 10 novices (*n* = 30, who had performed <10 simulator/endovascular procedures). All operators were right-handed. Each operator was asked to perform each of the five cannulation tasks three times in a randomized order. Each trial was considered as an independent test, thereby providing sufficient samples for comparison of the two distinct skill sets. Before commencing the study, all operators underwent a short training session in order to familiarize themselves with the use of the sensor. Appropriate endovascular tools, including guidewires with different stiffnesss, were provided to all operators.

#### Assessment of Haptic and Navigation Cues

The operator’s force and torque patterns can vary significantly, depending on the location of the catheter within the vasculature. Therefore, the procedure path for each of the cannulation tasks was divided into different phases. For the abdominal aneurysm model, the procedure was divided into three phases: (a) advancement of the catheter through the tortuous left common iliac artery and in the abdominal aorta, (b) selection of the renal artery, and (c) cannulation of each of the renal arteries, as shown in Fig. 1e. For the aortic arch model with the aneurysm, each tasks consists of the following three phases: (a) traversing the descending aorta, (b) navigating the aortic arch, and finally (c) cannulation of each of the arch vessels, as depicted in Fig. 1f.

Using the proximal force and torque signals as well as the 3-DoF position information obtained from the EM sensor at the catheter tip, different performance metrics were extracted at each phase of the procedure, including: median and maximum tip velocity, median and maximum tip acceleration, smoothness of motion (corresponding to the change in slope of the tip displacement signal), number of peaks in catheter tip displacement (corresponding to the back and forth movements of the tip), 3D total catheter path length (corresponding to the efficiency of motion), mean proximal forces in each axial direction (push/pull), mean torques applied in each rotational direction (clockwise/counterclockwise), and procedure time. Differences between the two experience groups were assessed using the non-parametric Wilcoxon rank-sum significance test on all metrics over each phase of the procedure. A value of ($P < 0.05$) was considered statistically significant. In order to provide a baseline for future references, the range of forces and torques applied over all procedures for the two experience groups is also reported. All the processing and statistical analysis was performed in Matlab (The MathWorks Inc., MA, USA).

*DTW similarity cost*: To further assess the repeatability and similarity in performance among different experience levels, dynamic time warping (DTW) was used to allow for non-linear synchronization and temporal alignment of the force and torque signals, and to analyze signal similarities in one of the most and one of the least experienced operators over all five of the endovascular catheterization tasks. Each operator has repeated each of the tasks *N* times, resulting in a series of *N* recordings for each of the tasks: $\left\{ r^i\right\} _{1\le i \le N}$. Each recording consists of time-series force and torque signals $r^i =\left\{ f^i, t^i\right\}$ of length $\left| r^i\right|$. Using DTW, each signal was temporally aligned to the signal whose length was closest to the average length of all recordings: $\sum \nolimits _{i=1}^N \left| r^i\right| / N$. Each alignment consists of finding a path, within an allowable range of steps, that maximizes the local match between the two signals. The cost of alignment $C^i$ between each signal and the reference signal can therefore be a good indication of their similarity. The average similarity cost is then used as a measure to assess the repeatability of that operator:

1 $$\begin{aligned} \text {Average similarity cost} =\frac{\sum \nolimits _{i=1}^N C^i}{N} \; . \end{aligned}$$

### Distal Force Sensing

This section provides details of the force measurement platform for measuring distal contact forces resulting from catheter–vessel interactions, and using this information as a means of improved characterization of operator skill and surgical performance.

**Figure 2:**
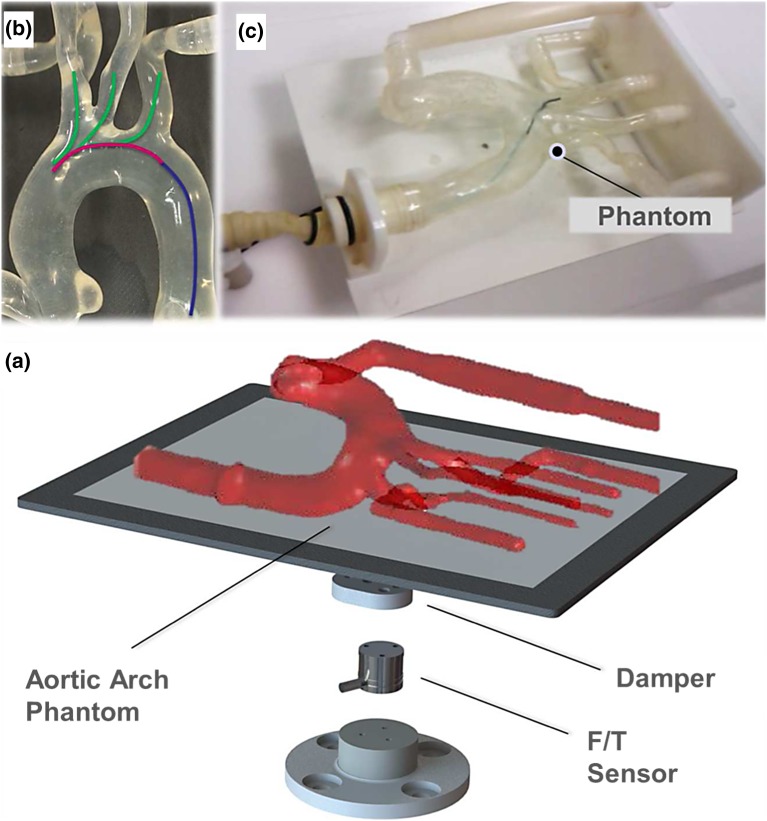
The distal force sensing platform with an exploded view of the F/T sensor (a), type I aortic arch model depicting the three endovascular tasks (b), and the experimental setup with the catheter within the model (c).

#### Distal Sensor Design

To provide direct measurement of the forces exerted on the vasculature, a distal force sensing platform was devised (Fig. 2).^16^ The platform consists of a silicone-based, transparent, anthropomorphic phantom (Elastrat Sarl, Switzerland), representing a type I aortic arch with bovine configuration of the left common carotid artery, mounted onto a plate and rigidly coupled to a 6-DoF F/T sensor (Nano17, ATI Industrial Automation, Inc., USA). The sensor provides force and torque readings in each of the three (X, Y, and Z) directions. In order to get an indication of the total forces exerted on the vascular model, an average root-mean-square (RMS) force modulus was calculated from the 3-DoF force measurements, in newtons (N). In order to obtain measurements that only result from direct contact between the catheter and the vascular model, the catheter was inserted through a custom-designed introducer sheath and a flexible section of tubing that decoupled the introducer sheath from the force sensing platform, as shown in Fig. 2c. The sensor was mounted close to the platform’s center of gravity, and an isolation damper was placed between the sensor and the plate to further remove any vibration artifacts.

#### Experimental Setup

As before, the processed video feed from a camera mounted above the vascular model was used as simulated 2D fluoroscopy guidance, and the above-mentioned custom software was used for synchronized acquisition of video and force date. The F/T measurements were read into LabVIEW at a frequency of 25 Hz and a resolution of 4 mN. In order to eliminate the weight of the platform and model, and ensure that the readings only correspond to the contact between the catheter and the model, the F/T sensor was zeroed at the beginning of each procedure.

Three endovascular tasks were defined for this study: cannulation of the LSA, the LCCA and the RCCA of the type I aortic arch phantom. Cannulation of each target vessel was performed multiple times (*n* = 42) across 14 operators from two experience groups: 4 experts (*n* = 12, experienced endovascular specialists who had performed >300 endovascular procedures), and 10 novices (*n* = 30, who had performed <10 endovascular procedures). Each operator was asked to cannulate each of the arch vessels three times in a randomized order, with each run considered as an independent trial, resulting in a total of 126 cannulations for all target vessels. Operators were provided with appropriate endovascular tools including a conventional 5F shaped catheter and Terumo guidewires.

#### Assessment of Skill

The procedure path for each of the three cannulations tasks was divided into three phases: a) traversing the descending aorta, b) moving through the aortic arch, and c) cannulation of each of the target arch vessels, as depicted in Fig. 2b. In order to remove any force bias and capture only tool–tissue interactions, the RMS force modulus recorded for each run was post-processed by subtracting all forces from the minimum recorded force value for that run (corresponding to non-tissue interaction). From the resulting force signal, different performance metrics were extracted for each phase of the procedure, including: mean, median, and maximum force values, force impact over time (**FIT**) (calculated by measuring the area under the force signals, corresponding to the force-time integral), standard deviation of forces, and number of force peaks (chosen to correspond to the number of significant contacts between the catheter and the arterial wall above a threshold of **1 N**). Differences between the two experience levels were assessed using the non-parametric Wilcoxon rank-sum significance tests. A value of ($P < 0.05$) was considered statistically significant. All data processing and statistical analysis was performed with the Matlab software (The MathWorks Inc., MA, USA).

*SVM binary classification of skill level*: To further validate the hypothesis that contact force measurements during endovascular intervention contain distinguishable patterns that are characteristic of skill, SVM-based binary classifiers were trained on the force signals in order to assess skill level for the different catheterization tasks. In order to obtain constant-size feature vectors across different runs, and assess the performance of the trained SVMs for better skill classification, the 1D force signals were sampled at regular intervals for different sample sizes *n* ($n = \{32, 64, 128\}$) by sampling the signals (length *T*) every *T*/*n* data points. Based on the 42 observation sequences for each of the 3 cannulation tasks, this resulted in fixed-size feature vectors *f* of ($n \times 42$), with known class labels for expert and novice for each of the trained SVM models.

From these feature vectors, binary SVM classifiers with radial basis function (RBF) kernels were trained for each of the catheterization tasks, for experts vs. novice classification with their corresponding known class labels. Using this kernel function, SVM classification nonlinearly maps the input observations into a higher dimensional space and finds a separating hyperplane with maximum margin between the two classes through an optimization step. The optimum parameters for the SVM model were estimated by performing a grid-search, and the performance of the classifier was evaluated through the ***k-fold cross-validation*** technique by dividing the training set into *k* subsets, and sequentially testing each left-out subset on the classifier trained from the remaining $k-1$ subsets. Common measures of performance corresponding to *precision*, *recall*, and *accuracy* were then calculated for each of the SVMs:

2 $$\begin{aligned} {\text{Precision}} = t_p/(t_p+f_p) \; . \end{aligned}$$

3 $$\begin{aligned} {\text{Recall}} = t_p/(t_p+f_n) \; . \end{aligned}$$

4 $$\begin{aligned} {\text{Accuracy}} = (t_p+t_n)/(t_p+t_n+f_p+f_n) \; . \end{aligned}$$

where $t_p$ is the number of true positives (experts classified as experts), $t_n$ is the number of true negatives (novices classified as novices), $f_p$ is the number of false positives (novices classified as experts), and $f_n$ is the number of false negatives (experts classified as novices).

## Results

### Proximal Force Sensing

Table 1 shows the results for the non-parametric test with median values for statistically significant differences between the two experience levels at each phase of the procedure, for cannulation of the two arteries in the abdominal model, and the three arteries in the thoracic model. Significant differences in force/torque patterns and catheter tip motion can be seen for cannulation of each of the arteries.

**Table 1:** Median values for statistically significant differences ($P < 0.05$) between proximal force metrics for expert (*n* = 18) vs. novice (*n* = 30) cannulation of each of the LRA and RRA of the abdominal model, as well as the LSA, LCCA, and RCCA of the thoracic model, at different phases of the procedure.

	Phase	Metric	Expert	Novice
LRA	Aorta	Med. speed (mm /s)	2.08 (1.7–2.8)	3.77 (2.2–8.0)
		Med. accel. $({\text{mm}}/{\text{s}}^2)$	44.3 (27.2–61.1)	92.56 (45.9–203.9)
		Smoothness $({\text{mm}}/{\text{s}}^2)$	2.1e5 (1.7e5–2.8e5)	4.4e5 (3.1e5–6.4e5)
		No. peaks	43 (38–62)	87 (45–114)
		Path length (mm)	414.4 (354.3–453.8)	613.4 (490.8–963.7)
		Pull force (N)	0.01 (0.007–0.04)	0.05 (0.02–0.11)
		Torque-CW (N mm)	0.27 (0.12–0.32)	0.44 (0.35–0.94)
	Artery selection	No. peaks	20 (15–40)	56 (3–108)
	Cannulation	Torque-CCW (N mm)	0.17 (0.04–0.30)	0.57 (0.12–1.37)
		Torque-CW (N mm)	**0.22** $^*$ (0.11–0.24)	0.56 (0.36–0.83)
RRA	Aorta	Med. speed (mm/s)	1.92 (1.7–2.5)	4.13 (2.5–7.5)
		Path length (mm)	421.49 (377.6–506.1)	613.75 (473.5–906.1)
		Pull force (N)	**0.01** $^*$ (0.005–0.3)	0.05 (0.03–0.11)
		Torque-CCW (N mm)	0.16 (0.08–0.28)	0.42 (0.13–1.01)
		Torque-CW (N mm)	**0.23** $^*$ (0.12–0.33)	0.53 (0.33–0.80)
	Artery selection	Max. speed (mm/s)	359.8 (265.9–459.8)	551.4 (333.7–735.0)
		Max. accel. (mm/s)	9.7e3 (6.5e3–1.3e4)	1.5e4 (1.0e4–1.8e4)
		Push force (N)	0.08 (0.03–0.45)	0.60 (0.28–0.93)
	Cannulation	Push force (N)	0.60 (0.24–0.98)	1.12 (0.81–1.60)
		Pull force(N)	0.02 (0.005–0.03)	0.11 (0.04–0.23)
		Torque-CW (N mm)	0.10 (0.05–0.39)	0.54 (0.32–0.63)
LSA	Aortic arch	Max. speed (mm/s)	**132.1** $^*$ (112.2–197.3)	421.5 (223.0–596.7)
		Max accel. $({\text{mm}}/{\text{s}}^2)$	**4.0** e3$^*$ (3.4e3–4.4e3)	9.9e3 (5.5e3–1.6e4)
		Smoothness $({\text{mm}}/{\text{s}}^2)$	**3.6** e4$^*$ (2.3e4–5.2e4)	2.5e5 (7.5e4–5.8e5)
		No. peaks	8 (5–23)	58 (16–96)
		Path length (mm)	**77.1** $^*$ (54.3–161.7)	505.4 (182.0–863.6)
		Pull force (N)	0.02 (0.003–0.09)	0.13 (0.04–0.30)
		Torque-CW (N mm)	**0.26** $^*$ (0.10–0.40)	0.89 (0.47–1.19)
		Time (s)	8.90 (4.1–19.3)	23.90 (11.1–52.6)
	Arch vessel	Pull force (N)	0.03 (0.02–0.08)	0.18 (0.08–0.37)
		Torque-CCW (N mm)	0.16 (0.11–0.46)	1.17 (0.71–2.28)
LCCA	Descending aorta	Push force (N)	**0.53** $^*$ (0.37–0.60)	0.73 (0.59–1.02)
		Pull force (N)	0.02 (0.01–0.06)	0.06 (0.04–0.14)
	Aortic arch	Pull force (N)	0.04 (0.01–0.08)	0.13 (0.08–0.20)
		Torque-CCW (N mm)	**0.39** $^*$ (0.10–0.55)	1.20 (0.84–1.62)
		Torque-CW (N mm)	0.46 (0.24–0.63)	0.61 (0.39–1.02)
		Time (s)	29.19 (25.5–53.3)	52.10 (33.5–77.1)
	Arch vessel	Torque-CCW (N mm)	**1.26** $^*$ (0.86–1.64)	2.50 (2.02–2.92)
RCCA	Descending aorta	Pull force (N)	0.02 (0.01–0.05)	0.06 (0.03–0.15)
		Torque-CW (N mm)	0.31 (0.16–0.48)	0.45 (0.29–0.84)
	Aortic arch	Pull force (N)	0.05 (0.03–0.07)	0.07 (0.06–0.17)

Metrics with $P < 0.001$ are indicated with a ($^*$). The interquartile range is shown in brackets

**Figure 3:**
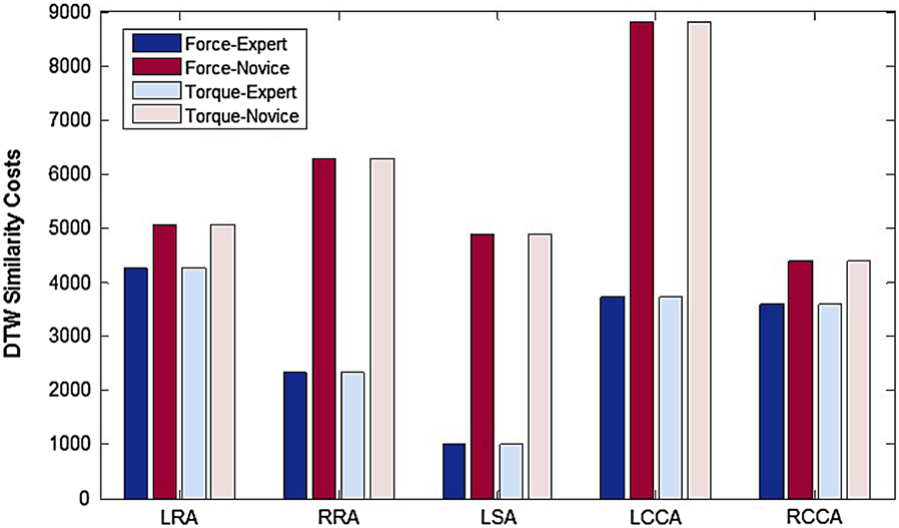
Average similarity costs, obtained through DTW for each of the force and torque signals, between expert and novice operator for cannulation of each of the five arteries.

Figure 3 shows the average similarity cost calculated through DTW, between one of the most and one of the least experienced operators, for the force and torque signals over multiple runs of each of the five catheterization tasks.

**Figure 4:**
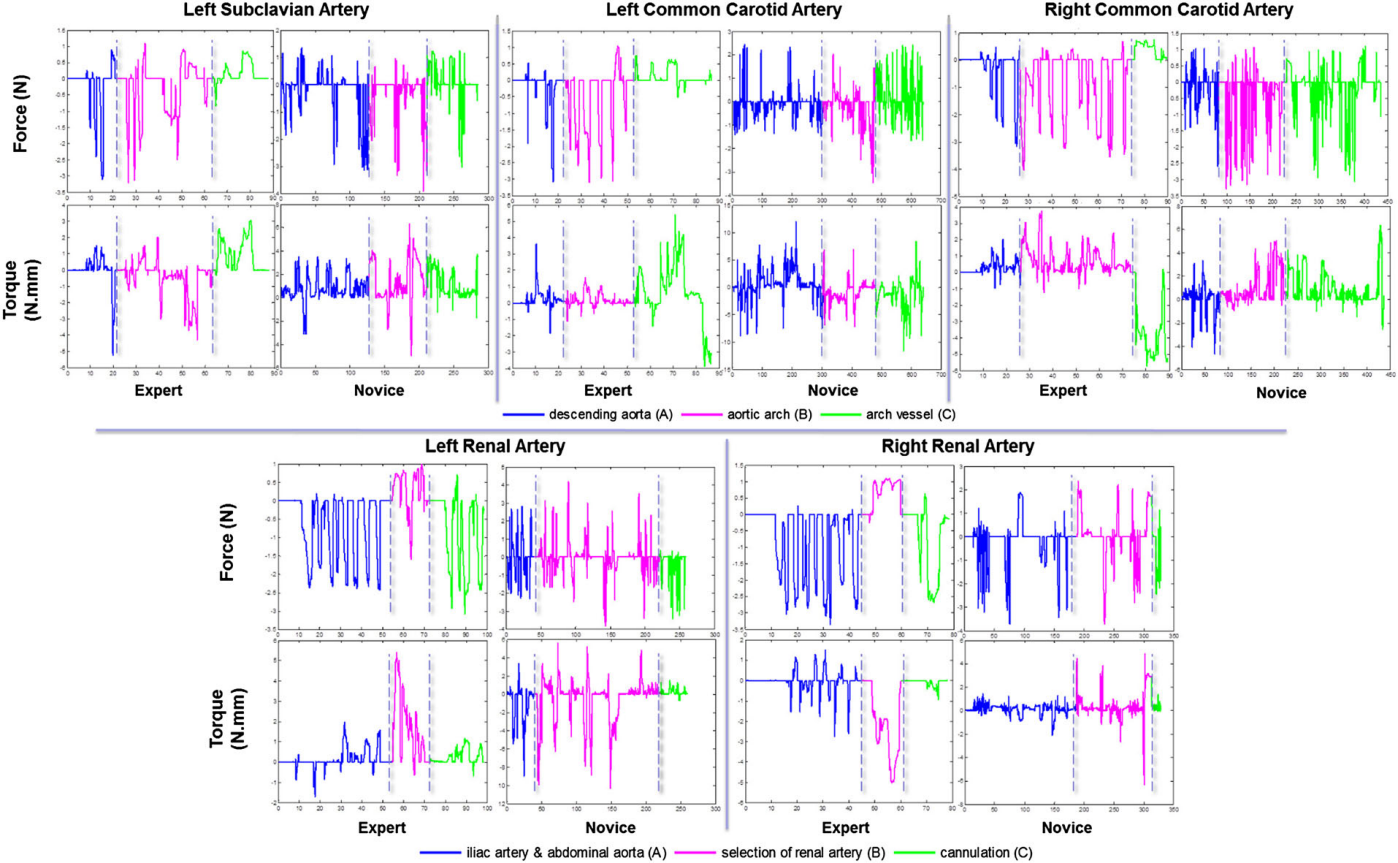
Examples of proximal force and torque signals for experienced vs. inexperienced operators, for cannulation of each of the five arteries. The three procedural phases are depicted in different colors.

Figure 4 shows examples of the force and torque plots for experienced vs novice operators, for cannulation of the LSA, the LCCA and the RCCA of the aortic arch model with aneurysm, and cannulation of the LRA and RRA in the abdominal aneurysm model. The plot shows significant differences in the force/torque patterns between the two experience levels, whilst highlighting the distinct and repeatable patterns of experienced operators across the different phases of the procedure.

Table 2 shows the means of the maximum force values (over all runs) in the axial direction, as well as the maximum torque values in each rotational direction. While depicting lower forces exerted by experienced operators for all three phases of the procedure (for all arteries), the results also show the higher reliance on torque for maneuvering through certain high-risk procedural phases such as the aneurysm (phase A) in the aortic arch.

**Table 2:** Means of the maximum force and torque values between expert and novice operators over the three phases, for each of the 5 cannulation tasks.

		Phase A		Phase B		Phase C	
		Exp.	Nov.	Exp.	Nov.	Exp.	Nov.
LRA	Max force (N)	2.57	2.64	1.24	2.09	3.01	3.14
	Max torque (N mm)	1.82	2.89	2.13	3.03	1.39	3.29
RRA	Max force (N)	2.39	2.60	0.82	1.52	1.66	2.63
	Max torque (N mm)	1.67	2.77	3.40	4.37	1.34	2.42
LSA	Max force (N)	1.68	1.98	1.51	2.22	1.89	2.03
	Max torque (N mm)	4.24	2.21	1.70	3.76	2.68	4.41
LCCA	Max force (N)	1.56	1.90	2.56	2.68	1.77	1.74
	Max torque (N mm)	3.86	2.55	2.62	5.26	5.52	6.77
RCCA	Max force (N)	1.83	1.90	2.60	2.99	1.75	2.26
	Max torque (N mm)	2.98	2.58	3.71	3.91	5.56	6.52

### Distal Force Sensing

Table 3 shows the differences in performance between expert and novice catheterization for cannulation of the of the LSA, LCCA, and RCCA respectively, by depicting the median values for statistically significant differences between the distal force metrics at each procedural phase.

**Table 3:** Median values for statistically significant differences ($P < 0.05$) between distal force metrics for expert (*n* = 12) vs. novice (*n* = 30) cannulation of each of the three LSA, LCCA, and RCCA arteries at different phases of the procedure.

	Phase	Metric	Expert	Novice
LSA	Descending aorta	Median force (N)	0.03	0.07
	Arch vessel	FIT (N s)	0.63	1.47
LCCA	Descending aorta	Median force (N)	0.04	0.06
	Arch vessel	Mean force (N)	0.11*	0.39
		Median force (N)	0.10*	0.20
		Max. force (N)	0.54*	1.75
		FIT (N s)	1.71*	9.11
		STDEV (N)	0.09*	0.42
		No. peaks	0*	12
RCCA	Descending aorta	Median force (N)	0.03	0.05
	Arch vessel	Mean force (N)	0.13*	0.24
		Median force (N)	0.07*	0.13
		Max force (N)	0.84*	1.40
		FIT (N s)	2.23	3.26
		STDEV (N)	0.12*	0.3
		No. peaks	0*	4

Metrics with $P < 0.001$ are indicated with a ($^*$)

**Figure 5:**
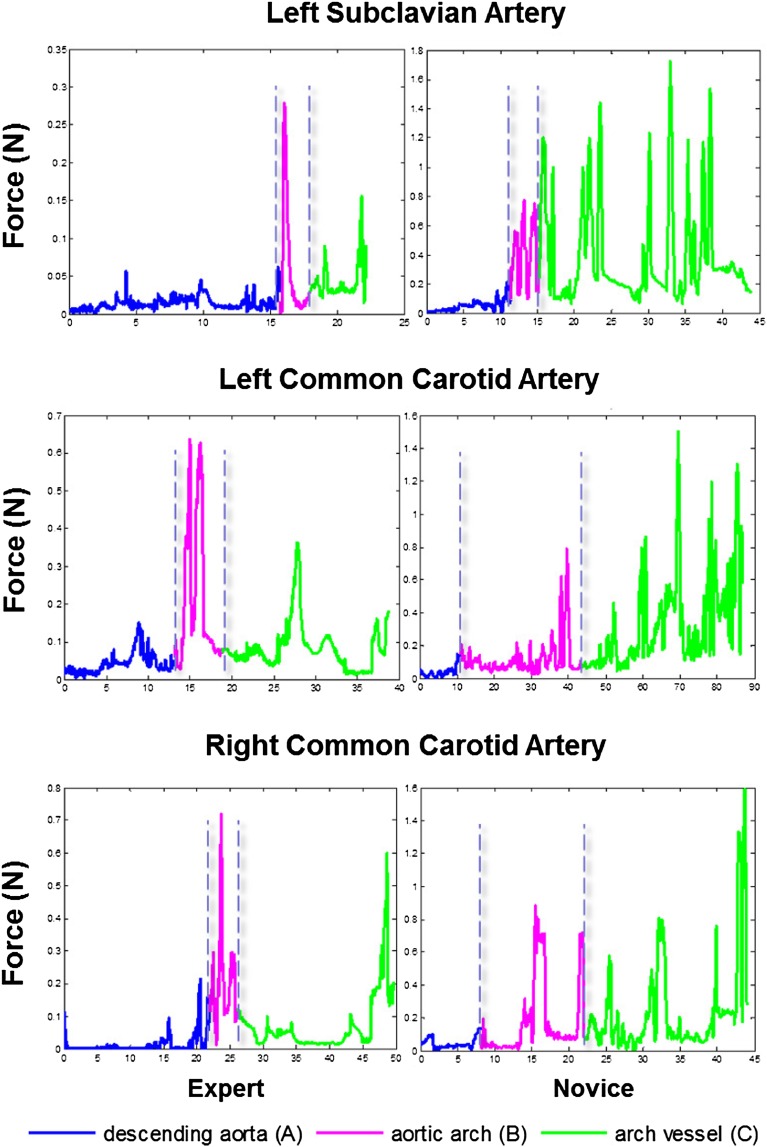
Examples of distal contact forces for experienced vs. inexperienced operators, for cannulation of each of the LSA, LCCA and RCCA. The three procedural phases are depicted in different colors.

Figure 5 shows examples of the distal contact forces for experienced vs novice operators, for cannulation of the LSA, the LCCA and the RCCA of the type I aortic arch phantom. As shown in the figure, significant differences can be seen in the magnitude, duration and repeatability between the forces exerted by each of the skill levels.

Table 4 shows the result of the SVM classification performance for the three cannulations tasks (LSA, LCCA, RCCA) for expert vs. novice skill level. The feature vectors *f* were created using different sampling sizes $n = \{32, 64, 128\}$, and the SVM classifiers were trained from the feature vectors and their corresponding class labels using the LIBSVM library.^1^ The ***k-fold cross-validation*** was run 10 times by changing the left-out sequence randomly, and the precision, recall and accuracy measures were calculated. The results show 82.5% classification accuracy for the LSA, 90% classification accuracy for the LCCA, and a classification accuracy of 90% for the RCCA with $n = 32$ sample size, corresponding to the best classification performance for each of the three tasks (highlighted in bold in the table).

**Table 4:** Binary SVM classification performance (RBF kernel) for expert vs. novice trials for different feature vector sampling sizes (*n*).

	*n*	Accuracy (%)	Precision (%)	Recall (%)
LSA	32	**82.5**	75.0	50.0
	64	80.0	80.0	67.0
	128	80.0	83.3	41.7
LCCA	32	**90.0**	90.0	75.0
	64	90.0	90.0	75.0
	128	87.5	81.8	75.0
RCCA	32	**90.0**	76.9	83.3
	64	87.5	81.8	75.0
	128	82.5	71.4	83.3

## Discussion

The results of this study offer significant insights into underlying force/torque patterns of operators and experience related skills that affect the quality and success of catheter navigation, as well as the forces exerted on the vasculature. The data depict significant difference in catheter tip velocities and accelerations, smoothness of catheter motion and number of back/forth movements, total catheter path length, the mean and maximum forces exerted on the vasculature, the force impact over time and the catheter contact with the arterial wall, between experienced and novice operators.

For the proximal force sensing platform, the results show significant differences in all performance metrics (tip velocity and accelerations, smoothness of motion, back and forth movements, total catheter path length, proximal forces and torques applied to the catheter) between experienced and novice operators, as shown in (Table 1. For the abdominal model, the differences are more evident in phase a, passing through the tortuous iliac artery and abdominal aorta with the aneurysm, which was the more anatomically challenging phase for this model. The results depict smoother and more stable catheter tip motion at lower speeds and accelerations and with reduced number of back and forth movements, combined with reduced forces and twists at the proximal end, for experienced operators. The results for the thoracic model depict stronger differences between the two experience groups when passing through high-risk areas such as going around the aortic arch and arch vessel cannulation (phases b and c). Experienced operators achieve safer and more stable catheter navigation at lower speeds and accelerations with reduced back and forth movements and a reduction in total catheter path length, while relying on lower pull forces (retractions) and reduced twists at the proximal end. Smoother tip movements and a reduction in total path length could potentially translate into reduced vessel wall contact and therefore less risk of dissection, perforation, or thrombosis, particularly in the presence of diseased vessels, as well as reduced potential of embolization and risk of stroke. The lower similarity costs obtained with the experienced operator for all five tasks (Fig. 3), further confirm the more repeatable and controlled execution patterns of experts against the difficulties encountered by novices, while performing repeated runs of the same procedure. Furthermore, the range of forces and torques exerted over all procedures for the two skill sets, as reported in Table 2, provides a valuable baseline for future studies on operator behaviour patterns and manipulation strategies. It should be noted that the forces applied by the operator include forces to overcome friction from the introducer sheath as well as the vasculature.

The results for the distal force sensing platform (Table 3) demonstrate better performance for all metrics (mean, median and maximum forces, FIT, standard deviation of forces, number of significant contacts) for experienced operators during cannulation of different target arch vessels. Force values are significantly lower for experienced operators for most procedural phases, for all three arteries. Significant improvements can also be seen in other metrics such as FIT, standard deviation of forces, and number of significant contacts, particularly during the vessel cannulation which is the more anatomically challenging procedural phase. More highlighted differences can be seen for cannulation of the LCCA, which for this model was the most difficult vessel to cannulate. The high accuracies of the binary SVM classification between expert and novice skill levels using the distal force measurements further validate the hypothesis that contact force measurements can contain distinct patterns of skill, with further applications towards improved clinical training and assessment of endovascular skill in a more objective, automated way. The results seem to indicate that distinguishable patterns of skill are captured better across smaller feature vector sample sizes, resulting in better classification between the two skill sets. Better classification performance is also seen for the more complicated LCCA and RCCA, compared to the LSA, for the same reason that these complex maneuvers can be more indicative of skill level.

### Limitations

While the proximal force measurement platform proposed in this study was validated on anthropomorphic phantoms representing different abdominal and aortic anatomies with different clinical complications, the distal force sensing platform was custom designed for the anthropomorphic phantom of the type I aortic arch with bovine configuration. The results showed significant differences between the two skill sets for cannulation of all three arch vessels of the model, however further studies on phantoms with different anatomies and clinical complications will provide more insights into the forces exerted on the vasculature when navigating through clinically challenging anatomies. Furthermore there are inherent limitations associated with the use of *in vitro* phantoms with water, by not reflecting the same biomechanical properties as real-tissue and the clinical complications associated with catheter navigation through tortuous iliac arteries, abdominal and aortic aneurysms, and atheroscelerotic aortic arches.

While the metrics in this study were designed to provide accurate, objective, and quantitative analysis of catheter motion stability, operator force interactions and technical performance, they cannot substitute real clinical outcomes. However they do provide significant insights on accuracy, control and technical skill towards successful catheter navigation. Further studies with phantoms utilizing blood-mimicking fluids as well as *ex vivo* porcine and cadaver tissue are encouraged with the proposed platforms, to provide more realistic biomechanical properties that are closer to the clinical setting.

### Conclusion and Future Work

The correct training of guidewire and catheter manipulation skills, based on an understanding of the forces exerted on the vasculature and the operator’s reaction to these while advancing the tools, is an important factor that has to be considered in endovascular skill training, in order to prevent damages caused by the interactions between the tools and the arterial wall, and the overexertion of force. Therefore a realistic simulation environment that enables objective and quantitative measurement of operator tool and tissue interactions can significantly improve clinical training of endovascular skill. The results reported in this paper depict significant differences between experienced and novice operators in the exerted force and torque patterns, catheter motion quality, as well as the magnitude and duration of contact forces, and highlight the importance of experience related skills that affect the overall success of catheterization.

The force sensing platforms and the proposed metrics have further potential to be used with *ex vivo* porcine or cadaver tissue to assess the safety of tissue manipulation based on the magnitude of forces that are exerted, and thereby provide criteria for relating the applied forces to the risk of tissue damage within different parts of the endovascular anatomy. The proximal force sensing platform can easily be miniaturized to become less obtrusive, and made with sterilizable material, to further facilitate the ease of use in clinical settings. The study proves the potential for proximal and distal force sensing as objective measures for endovascular skill assessment whilst providing significant insights into designing improved metrics for training of catheterization skills. Besides SVMs, the use of other machine learning approaches (e.g., HMMs) can further be explored for improved classification of skill in a non-binary sense.^16^ The outcome of this research has further potential towards providing design specifications for optimized development of future robotic catheter navigation systems that utilize the natural skills of operators,^14^,^15^ as well as evaluating the advantages of robot-assisted catheterization and assessment of robotic skill.^13^,^19^
